# Nerve and Vascular Biomarkers in Skin Biopsies Differentiate Painful From Painless Peripheral Neuropathy in Type 2 Diabetes

**DOI:** 10.3389/fpain.2021.731658

**Published:** 2021-10-22

**Authors:** Pallai Shillo, Yiangos Yiangou, Philippe Donatien, Marni Greig, Dinesh Selvarajah, Iain D. Wilkinson, Praveen Anand, Solomon Tesfaye

**Affiliations:** ^1^Diabetes Research Unit, Sheffield Teaching Hospitals NHS Foundation Trust, Sheffield, United Kingdom; ^2^Peripheral Neuropathy Unit, Hammersmith Hospital, Imperial College London, London, United Kingdom; ^3^Academic Unit of Radiology, University of Sheffield, Sheffield, United Kingdom

**Keywords:** pain, biomarkers, skin, vascular, painful diabetic neuropathy, von Willebrand Factor, IENFD, type 2 diabetes

## Abstract

Painful diabetic peripheral neuropathy can be intractable with a major impact, yet the underlying pain mechanisms remain uncertain. A range of neuronal and vascular biomarkers was investigated in painful diabetic peripheral neuropathy (painful-DPN) and painless-DPN and used to differentiate painful-DPN from painless-DPN. Skin biopsies were collected from 61 patients with type 2 diabetes (T2D), and 19 healthy volunteers (HV). All subjects underwent detailed clinical and neurophysiological assessments. Based on the neuropathy composite score of the lower limbs [NIS(LL)] plus seven tests, the T2D subjects were subsequently divided into three groups: painful-DPN (*n* = 23), painless-DPN (*n* = 19), and No-DPN (*n* = 19). All subjects underwent punch skin biopsy, and immunohistochemistry used to quantify total intraepidermal nerve fibers (IENF) with protein gene product 9.5 (PGP9.5), regenerating nerve fibers with growth-associated protein 43 (GAP43), peptidergic nerve fibers with calcitonin gene-related peptide (CGRP), and blood vessels with von Willebrand Factor (vWF). The results showed that IENF density was severely decreased (*p* < 0.001) in both DPN groups, with no differences for PGP9.5, GAP43, CGRP, or GAP43/PGP9.5 ratios. There was a significant increase in blood vessel (vWF) density in painless-DPN and No-DPN groups compared to the HV group, but this was markedly greater in the painful-DPN group, and significantly higher than in the painless-DPN group (*p* < 0.0001). The ratio of sub-epidermal nerve fiber (SENF) density of CGRP:vWF showed a significant decrease in painful-DPN vs. painless-DPN (*p* = 0.014). In patients with T2D with advanced DPN, increased dermal vasculature and its ratio to nociceptors may differentiate painful-DPN from painless-DPN. We hypothesized that hypoxia-induced increase of blood vessels, which secrete algogenic substances including nerve growth factor (NGF), may expose their associated nociceptor fibers to a relative excess of algogens, thus leading to painful-DPN.

## Introduction

Painful diabetic peripheral neuropathy affects up to a quarter of all patients with diabetes (1) and can lead to a significant curtailment of quality of life (2). Patients present with a range of sensory symptoms, including burning, aching and “electric shock-like” pains in their feet and legs (3). Night-time exacerbations of pain and contact hypersensitivity to bedclothes result in loss of sleep, and painful diabetic peripheral neuropathy (painful-DPN) can be disabling (4).

The pathophysiology of painful-DPN is complex, and there are no generally accepted disease-modifying treatments for the condition (4). The mainstay of treatment is symptom control with pharmacotherapy, which has limited efficacy and often dose-limiting side effects (5). Thus, there is a need to understand the underlying mechanisms of pain in DPN to help advance its treatment.

Intraepidermal nerve fiber (IENF) density in skin biopsies, using the pan-neuronal marker Protein Gene Product 9.5 (PGP9.5), is being used increasingly to diagnose small fiber neuropathy (6). Both animal and human studies have demonstrated a decrease in PGP 9.5 IENF density in DPN (7, 8). However, IENF density assessed with PGP9.5 is unhelpful in determining or predicting painful-DPN (9). Similarly, psychophysical tests, such as Quantitative Sensory Testing (QST) of small nerve fibers, may help diagnose DPN (10), but do not predict the presence of neuropathic pain (11). In recent studies, a more severe neuropathy was found in patients with painful-DPN (12, 13). Recent study has also implicated nerve fibers expressing calcitonin gene-related peptide (CGRP) (14, 15), and the nerve regeneration marker growth-associated protein 43 (GAP-43) (16, 17), in painful-DPN. Further, the vasculature may play a role, as reported in several studies. Increased sural nerve epineurial blood flow was found in subjects with painful-DPN (18). In subjects with treatment-induced painful neuropathy of diabetes, an abundance of epineural blood vessels, resembling the new vessels of the retina, was demonstrated (19). Impairment of microvascular reactivity in foot skin was linked to pain in diabetic neuropathy (20).

In this study, we have examined a panel of skin nerve and vascular markers in carefully characterized subjects with and without type 2 diabetes (T2DM) and compared DPN patients with and without neuropathic pain. We have used von Willebrand Factor (vWF), a vascular endothelial cell marker, to quantify dermal blood vessels (21).

## Materials and Methods

### Study Design and Participants

The study group consisted of 80 Caucasian subjects were included in this study: Healthy Volunteers (HV *n* = 19), and T2D subjects (*n* = 61), those without neuropathy (No-DPN, *n* = 19), with painless neuropathy (painless-DPN, *n* = 19), and with painful neuropathy (painful-DPN, *n* = 23). Patients were recruited from Sheffield Teaching Hospitals NHS Foundation Trust Diabetes Database and diabetes outpatient clinics. All patients had T2D diagnosed according to the WHO criteria (22). Exclusion criteria included non-diabetic neuropathies, history of alcohol consumption of more than 24 units a week, diabetic neuropathies other than distal symmetrical polyneuropathy, a peripheral vascular disease with absence of foot pulses, and neurological or other systemic disorders. All subjects gave written, informed consent before they participated in the study, which had Ethics Approval by the Sheffield Research Ethics Committee (Study Number STH15701).

### Neuropathy Assessment

All subjects underwent: (1) evaluation of the Douleur Neuropathique-4 (DN4) questionnaire (23); (2) Neuropathy Impairment Score Lower Limb [NIS(LL)] to assess clinical peripheral neurological status, (24); (3) Cardiac Autonomic Function Tests (CAFTs) using the O'Brien protocol, (25); (4) nerve conduction studies (NCS) of the sural, common peroneal, and tibial nerves at a stable skin temperature of 31°C and a room temperature of 24°C, using a Medelec electrophysiological system (Synergy Oxford Instruments, Oxford, UK); (5) detailed Quantitative Sensory Testing (QST), to assess large and small fiber function according to the German Research Network on Neuropathic Pain (DFNS) protocol (26), utilizing the Medoc TSA 2 Neurosensory analyser (Medoc Ltd., Ramat Yishai, Israel) (27); (6) sudomotor function assessment with measurement of foot Electrochemical Skin Conductance (ESC) using SUDOSCAN (Impeto Medical, Paris, France), as a marker of peripheral autonomic small fiber neuropathy (28). They also recorded average daily pain intensity scores for 7 days on an 11-point Numeric Pain Rating Scale (NPRS), ranging from 0 (no pain at all) to 10 (worst imaginable pain), from which an average daily pain score was calculated.

An overall Neuropathy Composite Score of NIS(LL)+7 was obtained by combining the NIS(LL) plus seven tests of nerve function (29). This is a validated, composite measure of neuropathy severity that has been used in epidemiological and population-based studies. Based on these assessments and the DN4 questionnaire score (12), participants with T2D were divided into three groups:

1) No-DPN, T2D consisting of asymptomatic participants with normal clinical and neurophysiological assessments.
2) Painless-DPN, comprising of pain-free participants with distal symmetrical polyneuropathy (1, 30).
3) Painful-DPN, participants with painful neuropathic symptoms involving the feet and/or legs in a distal symmetrical fashion together with evidence of peripheral neuropathy (1).

### Skin Biopsy

Skin biopsy specimens were obtained from the distal leg (10 cm above the lateral malleolus), in line with the guidelines published by the European Federation of Neurological Societies (EFNS) on the use of skin biopsy in the diagnosis of peripheral neuropathies (6). The skin was anesthetized by local infiltration of 2% lidocaine before a 3 mm punch biopsy was collected.

### Immunohistochemistry

The skin biopsy specimen was fixed for 12–18 h in paraformaldehyde-lysine-periodate and cryoprotected overnight (15% sucrose in.1M phosphate buffer) at 4°C, then snap-frozen in liquid nitrogen. Then, 15 μm tissue sections were collected with a freezing microtome for assessment of intra-epidermal (IENF) and sub-epidermal (SENF) nerve fiber density (30 μm thickness for GAP-43 to increase the sensitivity of this marker in the epidermis). Sections were incubated overnight with primary antibodies to the structural pan-neuronal marker PGP9.5 (1:40,000; Ultraclone, Isle of Wight, UK), the marker of regenerating nerve fibers GAP-43 (1:80,000; Sigma-Aldrich, Dorset, UK), the sensory neuropeptide CGRP (1:2,000; Novocastra Ltd, Newcastle Upon Tyne, UK), and to the vascular marker vWF (1:10,000; Novocastra Laboratories, Milton Keynes, UK). They were then detected using avidin-biotin-peroxidase methods (ABC; Vector Laboratories, Peterborough, UK) giving black, positive immunostaining as previously described, and validated for the section thickness used, i.e., 15 μm compared with 50 μm, the former section thickness enables a range of markers to be studied in the same biopsy (31–33). Tissue sections were counterstained for nuclei in 1% w/v aqueous neutral red. Omission of primary antibodies and sequential dilution of antibodies gave appropriate results for specificity.

Intraepidermal nerve fibers were counted along the length of four non-consecutive sections. The length of epithelium in each counted section was measured using computerized microscopy software (Olympus ANALYSIS 5 Soft, Olympus UK, Southend, Essex, UK), and results were expressed as fibers/mm length of the section. SENF was measured by image analysis, where digital photomicrographs were captured *via* video link to an Olympus BX50 microscope (Olympus Optical Co. Ltd., Tokyo, Japan) with a depth of 200 μm below the basal epidermis. The gray-shade detection threshold was set at a constant level to allow detection of positive immunostaining and the area of highlighted immunoreactivity was obtained as a percentage (% area) of the field scanned. Images were captured (×40 objective magnification) along the entire length and the mean values were used for statistical analysis. Quantification was performed by two independent blinded observers and there was no significant difference between observers.

### Statistical Analysis

The statistical package SPSS version 24 (SPSS, IBM Corp, NY, USA) was used for baseline data, and GraphPad Prism version 5 for Windows (GraphPad Prism Software, San Diego, CA, USA) for skin biopsy data. Baseline subject characteristics were described as mean and SDs for normally distributed variables, as the median and interquartile range for variables with a skewed distribution, and percentages for categorical variables. We have used Spearman's rank correlation for non-parametric variables. One-way ANOVA was used to compare the mean of baseline characteristics. Skin biopsy data were analyzed using Mann–Whitney U test and *p* < 0.05 (two-tailed) indicated significance.

## Results

Table 1 summarizes demographic details and study assessments performed for each group. The groups were matched for age and gender. The diabetes patient groups had a higher body mass index (BMI, ANOVA *p* < 0.01) compared with HV. There was no statistically significant difference in duration of diabetes and HbA1c among the diabetes groups. Table 2 shows clinical, neurophysiological, quantitative sensory testing, and skin biopsy data of the different groups. As expected, the DPN groups had significantly higher NISLL+7 scores compared with the No-DPN and HV groups. The painful-DPN group had a higher NISLL+7 score compared with the painless-DPN group (as reported previously). The DN4 score and 24-h numeric pain rating scale (NPRS) pain score was significantly higher in the painful-DPN group compared with all the other groups (*p* < 0.01). While warm detection thresholds (WDT) were significantly raised in both DPN groups (*p* < 0.01), with no differences between painful- and painless-DPN, cold detection threshold (CDT) was significantly reduced in the painful-DPN compared with painless-DPN (*p* < 0.01). There was also a significant increase in thermal sensory limen (TSL) in painful-DPN (*p* < 0.01). SUDOSCAN foot electrochemical skin conductance, a measure of autonomic sudomotor function, was significantly reduced in DPN groups compared with No-DPN and HV groups (Feet ESC, ANOVA *p* < 0.01) but *post-hoc* analysis showed no difference between painful and painless-DPN groups (Feet ESC, LSD *p* = 0.45). Calf skin IENFD and SENFD were severely diminished in both DPN groups, with no differences between the two.

**Table 1:** Demographic characteristics (mean/SD for normally distributed variables and median/interquartile range for nonparametric variables) of study subjects.

	**HV**	**No–DPN**	**Painless–DPN**	**Painful–DPN**	***p*** **Value ANOVA**
n	19	19	19	23	
Age (years)	55.1 (10.6)	58.3 (8.6)	60.8 (10.0)	60.2 (10.2)	0.27
Gender (male %)	47.3	52.6	68.0	56.5	0.61
BMI (kg/m^2^)	26.8 (4.2)	31.5 (6.1)	30.3 (5.2)	33.7 (5.4)	0.001
Diabetes duration (years)*	–	8.0 (6.5)	14.5 (16.2)	13.0 (19.5)	0.24
HbA1c (mmol/mol)	–	63.3 (20.0)	61.5 (8.0)	66.9 (13.8)	0.51
Urine ACR (mg/mmol)*	–	0.8 (0.9)	1.7 (7.2)	4.6 (22.3)	0.13
eGFR (mls/min/1.73m^2^)	–	73 (13.9)	68.1 (20.1)	61.6 (19.6)	0.30

*DPN, diabetic peripheral neuropathy; DM, diabetes mellitus, HV, healthy volunteers; BMI, body mass index; ACR, albumin creatinine ratio; GFR, glomerular filtration rate*.

* *non-parametric variables*.

**Table 2:** Clinical parameters of study subjects [mean(SD)].

	**HV**	**No-DPN**	**Painless DPN**	**Painful DPN**	***p*** **Value ANOVA**
NISLL+7	0.5 (0.8)	1.4 (1.2)	19.6 (7.6)	28.9 (16.4)	<0.01
DN4	0	0.1 (0.5)	2.1 (0.8)	5.9 (1.8)	<0.01
NPRS	0	0	0	7.06(1.7)	<0.01
CDT (°C)	27.1 (3.2)	25.0 (3.2)	19.5 (10.6)	11.2 (10.6)	<0.01
WDT (°C)	39.3 (3.6)	39.6 (3.5)	46.4 (3.9)	47.2 (3.0)	<0.01
TSL (°C)	13.6 (7.2)	17.09 (8.7)	30.9 (12.0)	37.9 (12.3)	<0.01
Feet ESC (μS)	80.7 (7.6)	70.5 (17.8)	45.3 (24.5)	50.3 (27.7)	<0.01
SNCV (m/s)	48.8 (11.1)	44.4 (6.5)	16.4 (20.7)	15.1 (19.1)	<0.01
Sural Amplitude (mA)	15.7 (7.5)	12.4 (5.6)	2.3 (3.2)	2.9 (3.6)	<0.01
CPCV (m/s)	46.7 (4.2)	43.4 (4)	37.6 (23.8)	23.6 (20.4)	<0.01
CP Amplitude (mA)	5.6 (1.9)	4.6 (1.9)	2.4 (2.3)	1.5 (1.7)	<0.01
Tibial Latency (ms)	4.2(0.9)	4.2 (0.6)	5.7 (2.8)	3.6 (3.7)	0.2
IENFD (fibers/mm)	5.6 (1.3)	5.3 (3.1)	0.63 (1.4)	0.82 (1.7)	<0·01
SENFD (% area)	1.0 (0.4)	1.6 (0.9)	0.35 (0.6)	0.14 (0.2)	<0.01

*DPN, diabetic peripheral neuropathy; DM, diabetes mellitus; HV, healthy volunteers; NISLL+7, Neuropathy Impairment Score of the Lower Limb plus 7 tests of nerve function; DN4, Douleur Neuropathique-4 painful neuropathy score; NPRS, Numeric Pain Rating Scale; IENFD, Intra Epidermal Nerve Fiber Density; SENFD, Sub Epidermal Nerve*.

*Fiber Density; CDT, Cooling Detection Threshold; WDT, Warm Detection Threshold; TSL, Thermal Sensory Limen; ESC, Electrochemical Skin Conductance; SNCV, Sural Nerve Conduction Velocity; CPCV, Common Peroneal Conduction Velocity; CP, Common Peroneal*.

### Protein Gene Product 9.5

**Figure 1:**
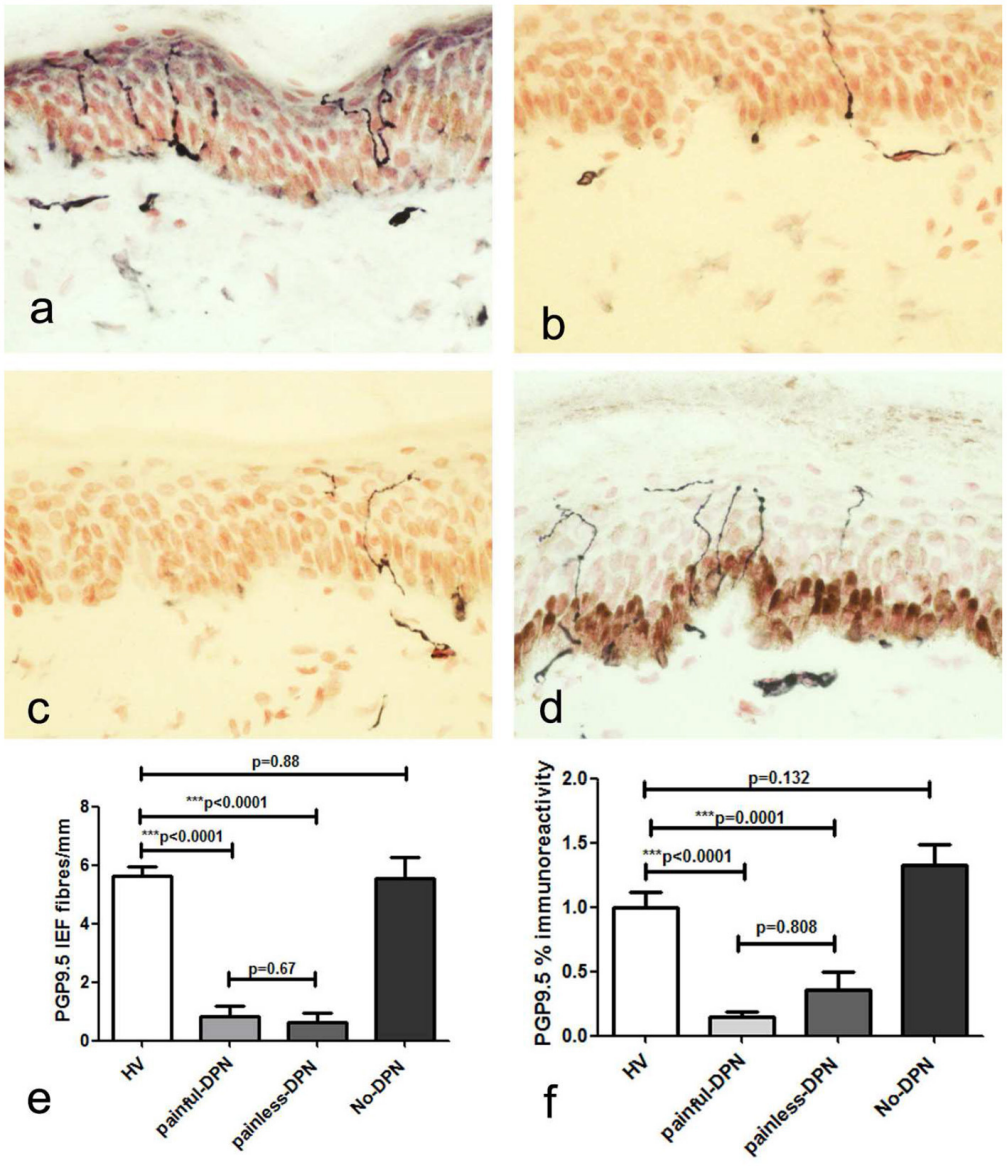
**(a)** Protein gene product 9.5 (PGP9.5) immunoreactivity in calfskin from healthy volunteers, HV, **(b)** painful-diabetic peripheral neuropathy (DPN), **(c)** painless-DPN, and **(d)** No-DPN. **(e)** Mean ± SEM of the PGP9.5 intra-epidermal fibers (fibers/mm). **(f)** Image analysis of PGP9.5 sub-epidermal counts (% immunoreactivity) in the same groups (HV, painful-DPN, painless-DPN, No-DPN).

Immunostaining for the pan-neuronal marker (PGP 9.5) in skin biopsies from the HV and No-DPN groups showed sub-epidermal nerve fascicles (SENF) adjacent to the basal epidermis, with IENF emerging from them (Figure 1). These were markedly and significantly reduced in both DPN groups, with pain or without pain (*p* < 0.0001). Image analysis of sub-epidermal PGP9.5-immunoreactive nerve fibers (SENF) in both painful-DPN and painless-DPN also showed a similar reduction compared to the HV group (SENF, *p* < 0.0001 and *p* = 0.0001, respectively). However, there was no statistical difference between HV vs. No-DPN groups, and painless-DPN vs. painless DPN groups, for both IENF and SENF analyses.

### Growth-Associated Protein 43

**Figure 2:**
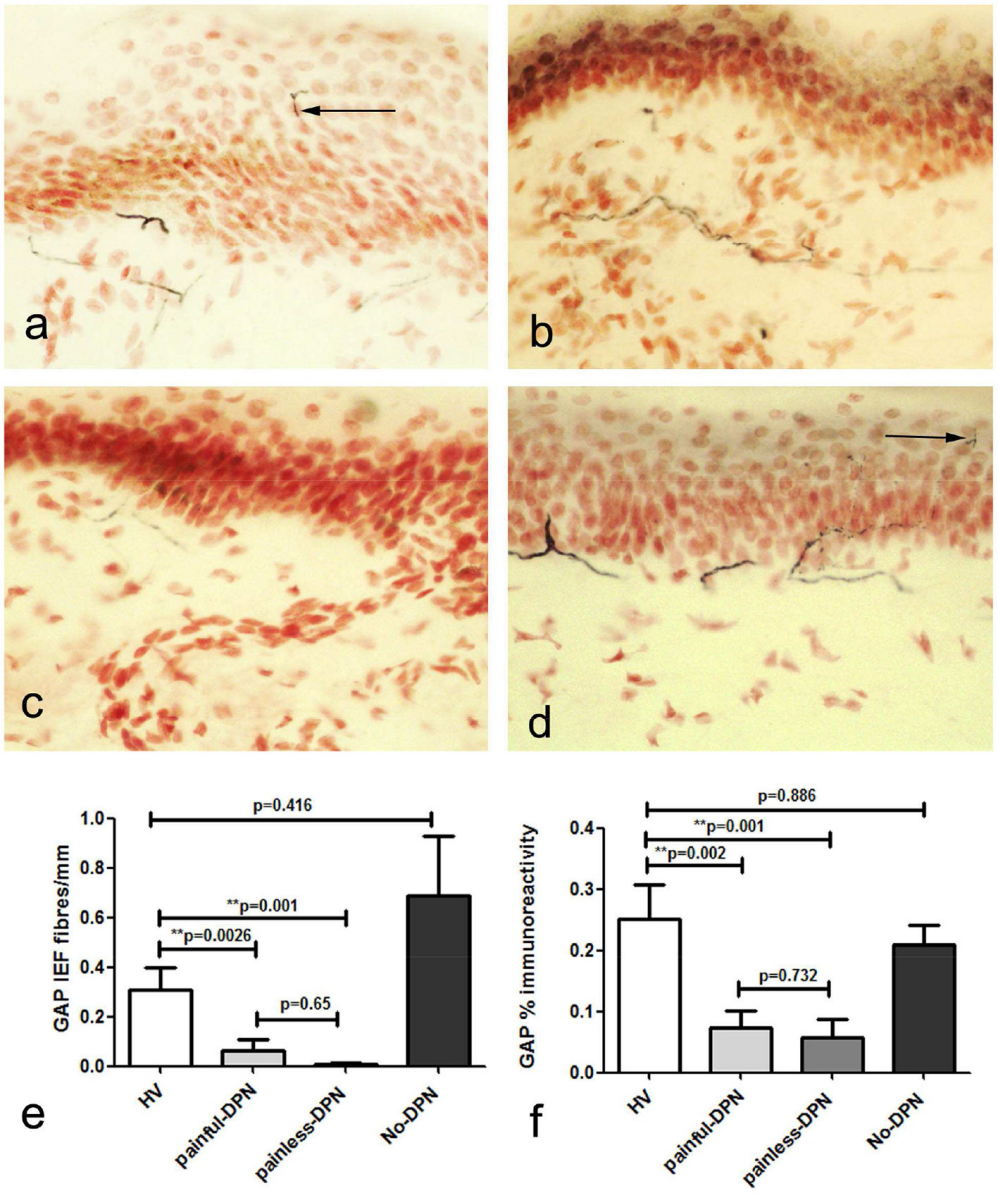
**(a)** Growth-associated protein 43 (GAP43) immunoreactivity in calfskin from healthy volunteers, HV, **(b)** painful-DPN, **(c)** painless-DPN, and **(d)** No-DPN. **(e)** Mean ± SEM of the GAP-43 intra-epidermal fibers (fibers/mm). **(f)** Image analysis of GAP43 sub-epidermal counts (% immunoreactivity).

Immunostaining for the nerve regeneration marker (GAP43) in skin biopsies from the HV group showed a similar pattern to PGP9.5 (Figure 2). GAP43 IENF density was significantly reduced in both painful-DPN (*p* = 0.0026) and painless-DPN groups (*p* < 0.001) compared with the HV group. Image analysis of sub-epidermal GAP43-immunoreactive nerve fibers (SENF) in painful-DPN and painless-DPN groups also showed a similar reduction compared to the HV group (SENF GAP-43, *p* < 0.002 and *p* = 0.001, respectively); again, there was no statistical difference between HV vs. No-DPN groups, or for painful-DPN vs. painless-DPN groups, for both IENF and SENF GAP43 analyses.

### Calcitonin Gene-Related Peptide

**Figure 3:**
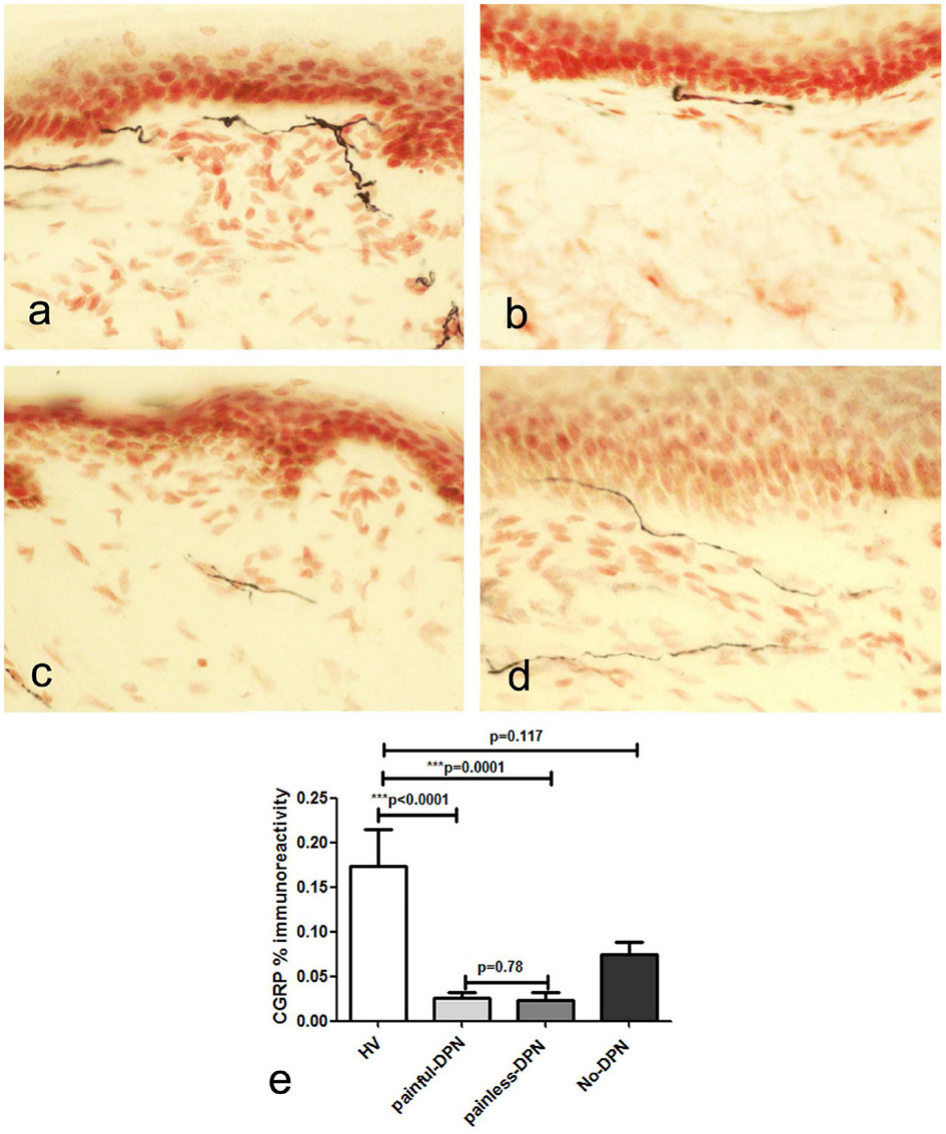
**(a)** Calcitonin gene-related peptide (CGRP) immunoreactivity in calfskin from healthy volunteers HV, **(b)** painful-DPN **(c)** painless-DPN, and **(d)** No-DPN. **(e)** Image analysis of CGRP sub-epithelial counts (% immunoreactivity) in the same groups (HV, painful-DPN, painless-DPN, no-DPN). Intra-epithelial fibers were absent or sparse in all groups.

Immunostaining for CGRP showed severely diminished IENF density, so only SENF were quantified (Figure 3). The CGRP image analysis also showed a similar decrease in both DPN groups (painful-DPN, *p* < 0.0001; painless-DPN, *p* = 0.0001) compared with the HV group. There was no statistical difference between the painful-DPN and painless-DPN groups for SENF CGRP analyses.

### Von Willebrand Factor

**Figure 4:**
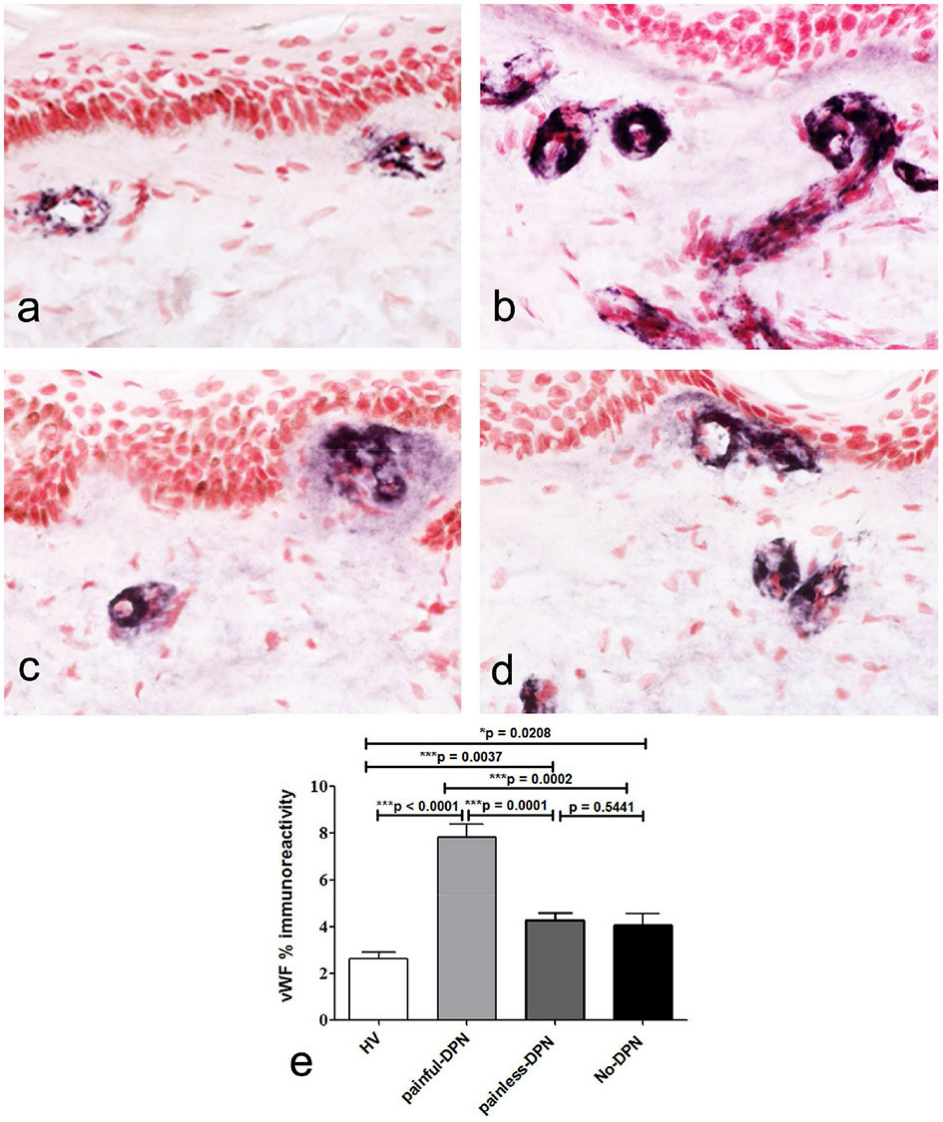
**(a)** von Willebrand Factor (vWF) immunoreactivity in calfskin from healthy volunteers, HV **(b)**, painful-DPN **(c)** painless-DPN, **(d)** No-DPN, **(e)** image analysis of vWF sub-epithelial endothelial staining (% immunoreactivity).

**Figure 5:**
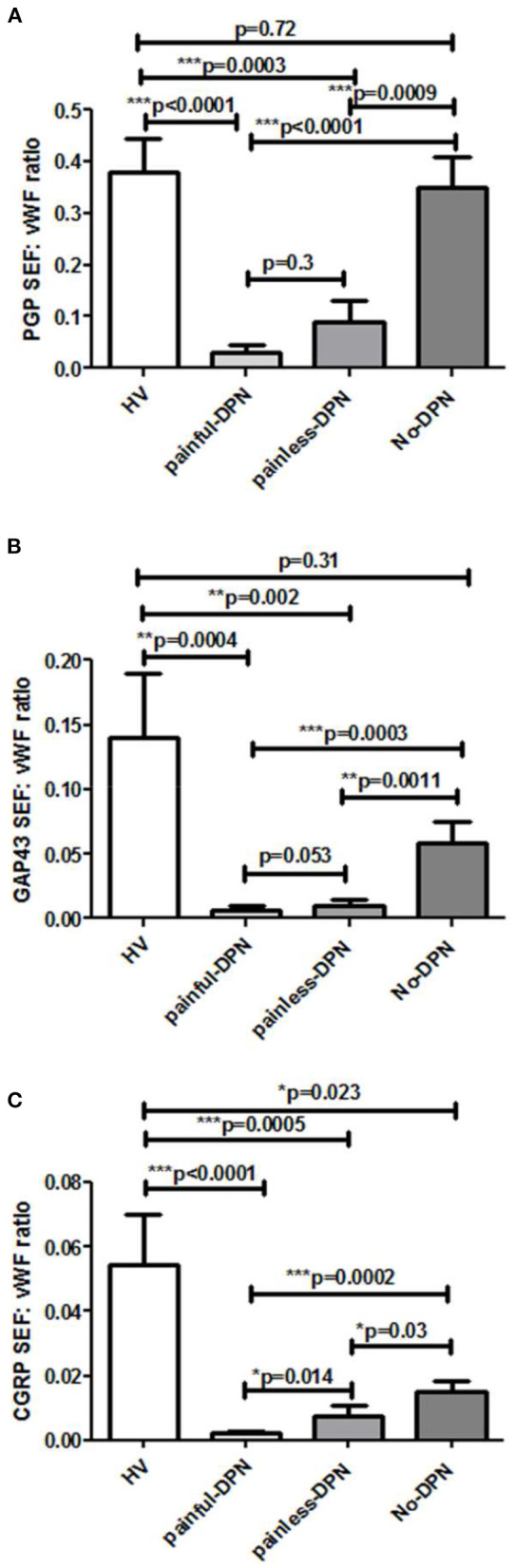
Bar charts showing image analyses (mean ± SEM) of **(A)** PGP9.5 sub-epidermal nerve fiber (SENF) (SEF): vWF, **(B)** GAP43 SENF: vWF, and **(C)** CGRP SENF: vWF ratios.

Immunostaining for the vascular marker, vWF, showed increased sub-epidermal staining area in all diabetes groups vs. the HV group (Figure 4). There was a highly significant increase in the vascular staining in both groups with DPN compared to the HV group (painful-DPN, *p* < 0.0001; painless-DPN, *p* = 0.0037), and also in the No-DPN group (*p* = 0.02), compared with the HV group. There was significant correlation of vWF to QST parameters especially to thermal thresholds, CDT and HPT (CDT; cc = −0.379, *p* < 0.004, HPT; *r* = 0.435, *p* = 0.002) (Table 3) and DN4 pain score (DN4; cc = 0.632, *p* < 0.0001). Expressing the results as a ratio of PGP9.5 (SENF) to vWF showed similar results in painful-DPN (*p* < 0.0001) and painless-DPN (*p* = 0.0003), but not with the no-DPN group (*p* = 0.72) (Figure 5A). Similarly, expressing the results as a ratio of GAP43 (SENF) to vWF showed similar results in the painful-DPN (*p* = 0.0004) and painless-DPN (*p* = 0.002) groups, but not with the No-DPN group (*p* = 0.31) (Figure 5B). Expressing the results as a ratio of CGRP (SENF) to vWF showed similar results (painful-DPN, *p* < 0.0001, painless-DPN, *p* = 0.0002), and a significant decrease with the No-DPN group (*p* = 0.02) (Figure 5C) compared with HV. The ratio of sub-epidermal nerve fiber (SENF) density of CGRP:vWF showed a significant decrease in the painful-DPN group vs. painless-DPN group (*p* = 0.014) (Figure 5C), but this was not significant for ratios of the other nerve markers.

**Table 3:** Correlation Coefficients of SE PGP, IE PGP, and combined IE/SE corrected vWF to QST parameters.

		**CDT**	**WDT**	**TSL**	**CPT**	**HPT**	**PPT**	**VDT**
vWF/*SE PGP	CC	−0.379	0.265	0.203	−0.168	0.435	0.093	0.209
	*P* value	**0.004**	**0.049**	0.134	0.216	**0.001**	0.494	0.121
vWF/*IE PGP	CC	−0.351	0.223	0.159	−0.133	0.395	0.116	−0.127
	*P* value	**0.008**	0.099	0.242	0.330	**0.003**	0.393	0.350
vWF/*IE and SE PGP	CC	−0.349	0.207	0.150	−0.125	0.395	0.102	−0.132
	*P* value	**0.009**	0.130	0.275	0.363	**0.003**	0.457	0.337

*vWF, von Willibrand factor; IE, Intra Epidermal; SE, Sub Epidermal; CDT, Cooling Detection Threshold; WDT, Warm Detection Threshold; TSL, Thermal Sensory Limen; CPT, Cold Pain Threshold; HPT, Heat Pain Threshold; PPT, Pressure Pain Threshold; VDT, Vibration Detection Threshold*.

* *covariates used in the analyses. IE, intraepidermal, SE, subepidermal. Significant results (p < 0.05) are shown in bold*.

## Discussion

In this study, we reported significant nerve fiber loss in distal leg skin biopsies from patients with DPN using PGP9.5, CGRP, and GAP43 nerve fiber immunostaining. This was apparent for IENF and SENF density in both painful-DPN and painless-DPN groups, compared with the HV group, and patients without the No-DPN group. In contrast, there was a significant increase in the vascular marker vWF in skin biopsies from all diabetes (T2DM) groups, which was particularly marked in the painful-DPN group. Importantly, this increase of blood vessels was significantly greater in the painful-DPN than the painless-DPN group. There was a significant correlation of vWF to thermal thresholds especially CDT and HPT also to DN4 pain score supporting vascular etiology in painful-DPN and predominant involvement of small fibers.

Our finding of similarly reduced PGP9.5 IENF density in painful-DPN and painless-DPN groups agrees with previous studies (16, 32, 34). The severity of IENF loss was indicative of advanced DPN in both our painful-DPN and painless-DPN groups. GAP43, the selective marker for regenerating nerve fibers, was decreased in DPN, as in previous studies (32, 34). Studies of skin biopsies from proximal thigh showed higher GAP43:PGP ratios in painful-DPN compared with painless-DPN subjects (16, 17). However, this was not observed in our cohort with advanced DPN in distal leg skin biopsies, and future studies are indicated in mild/early DPN.

Our further analyses, comparing nerve fiber density to the vascular marker, vWF, showed that CGRP to vWF ratio was lower in painful-DPN than in painless-DPN. Hence, we hypothesized that in advanced painful DPN, the increased dermal vasculature, and its ratio to reduced/surviving nociceptors, i.e., CGRP-expressing small sensory nerve fibers, may differentiate painful-DPN from painless-DPN. Increased blood vessels following tissue ischaemia and hypoxia, associated with disproportionate, i.e., relatively fewer, CGRP nociceptor nerve fibers, may lead to painful-DPN. While the % ratio of CGRP to vWF of no-DPN vs. HV was significantly reduced, this reduction was greater in DPN, particularly painful-DPN. These proposed mechanisms and the supportive evidence (35–38) were discussed below.

The density of IENF is known to be decreased in a wide range of neuropathic pain syndromes, including postherpetic neuralgia (PHN), painful-DPN, and painful HIV-associated neuropathy (38). Further, it is now widely accepted that the residual/surviving nociceptors may develop hyperexcitable properties, due to exposure to relatively high concentrations of Nerve Growth Factor (NGF) and other algogens (37, 38). We originally proposed that disproportionately high NGF levels in the target organ and its surviving nociceptors expressing its high-affinity receptor TrkA may underlie neuropathic pain (36). These surviving nociceptors may correspond to the later description of “irritable nociceptors” in PHN skin (39), or “over-trophed nociceptors”. In normal human skin, NGF is predominantly produced and secreted by keratinocytes and blood vessels (35, 37). NGF is up-regulated by inflammation, wherein nociceptor terminals in skin exposed to higher NGF become hyperactive and sprout, resulting in pain and hypersensitivity (36). These processes and interactions are more complex in different stages of painful neuropathies (37, 38). We had previously shown a decrease in CGRP nociceptors, which were regulated by nerve growth factor (NGF), and also reduced NGF expression in early DPN (35). However, a relative excess of NGF, so akin to the NGF to TrkA nerve fiber disproportion in inflammation, may sensitize residual CGRP nociceptors, and lead to neuropathic pain (36–38). Thus, at the early stage of DPN, the ratio of NGF in keratinocytes to residual dying-back IENF may underlie hypersensitivity. At the late stage of advanced DPN, with marked or complete loss of IENF as in this study, the increased ratio of blood vessels to associated dermal nociceptors (SENF), e.g., those which express CGRP, may contribute to the development of neuropathic pain. The algogens related to increased blood vessels may include NGF, secreted from the blood vessels themselves, as well as NGF and cytokines, secreted by Schwann and inflammatory cells, consequent to nerve degeneration (35–38). Future studies should include assessment of NGF levels and their potential role in angiogenesis, and contribution of local or circulating inflammatory cells, at different stages of painful-DPN.

We have reported similar changes in subjects with painful non-freezing cold injury (NFCI or Trench Foot). Distal leg skin biopsies from patients with chronic NFCI showed a marked increase of blood vessels, as indicated by the immunostaining for vWF and also vascular endothelial growth factor (VEGF). The increased vasculature may reflect the changes secondary to hypoxia and ischaemia, with the formation of new blood vessels, as indicated by increased VEGF (38), which in NFCI may be induced by cold and mechanical pressure. The ratios of nerve markers to vWF were also decreased in painful NFCI calfskin, as in painful-DPN in this study.

Our previous studies in patients with painful-DPN have reported involvement of the vasculature: increased peripheral nerve epineurial blood flow (18), altered foot skin microcirculation (20), increased thalamic vascularity (40), and autonomic dysfunction (41). Laser Doppler studies have shown altered capillary circulation in the feet of patients with DPN (42). Increased plasma vWF has been associated with neuropathic foot ulceration (43). In addition to the vasculature, thalamic neurotransmitter mechanisms (44) and metabolic factors such as Vitamin D (45) may also play a role in painful-DPN.

Our results indicated that further studies are required, particularly of underlying neuro-vascular mechanisms, which may help to improve our understanding of neuropathic pain development and its treatment.

## Data Availability Statement

The raw data supporting the conclusions of this article will be made available by the authors, without undue reservation.

## Ethics Statement

The studies involving human participants were reviewed and approved by Sheffield Research Ethics Committee. The patients/participants provided their written informed consent to participate in this study.

## Author Contributions

PS conducted the clinical study. YY and PD analyzed the tissues and all wrote the manuscript. MG, DS, and IW helped set up the study and contributed to the discussion. PA and ST conceived and supervised the study and wrote the manuscript. All authors contributed to the article and approved the submitted version.

## Funding

ST thanks Sheffield Teaching Hospitals Diabetes Charitable Trust for funding the study.

## Conflict of Interest

The authors declare that the research was conducted in the absence of any commercial or financial relationships that could be construed as a potential conflict of interest.

## Publisher's Note

All claims expressed in this article are solely those of the authors and do not necessarily represent those of their affiliated organizations, or those of the publisher, the editors and the reviewers. Any product that may be evaluated in this article, or claim that may be made by its manufacturer, is not guaranteed or endorsed by the publisher.

## Abbreviations

T2DM: type 2 diabetes
DPN: diabetic peripheral neuropathy
DM: diabetes mellitus
HV: healthy volunteers
NISLL+7: Neuropathy Impairment Score of the Lower Limb plus 7 tests of nerve function
DN4: Douleur Neuropathique-4 painful neuropathy score
NPRS: Numeric Pain Rating Scale
IENF: Intra Epidermal Nerve Fibre
SENF: Sub Epidermal Nerve Fibre
CDT: Cooling Detection Threshold
WDT: Warm Detection Threshold
TSL: Thermal Sensory Limen
ESC: Electrochemical Skin Conductance
BMI: body mass index
ACR: albumin creatinine ratio
GFR: glomerular filtration rate
PGP9.5: Protein Gene Product 9.5
GAP43: growth-associated protein 43
CGRP: calcitonin gene-related peptide
vWF: von Willebrand Factor.
